# Who is quitting? An analysis of the dis-adoption of climate smart sorghum varieties in Tanzania

**DOI:** 10.1108/IJCCSM-11-03-341

**Published:** 2019-05-20

**Authors:** Franklin Simtowe, Kai Mausch

**Affiliations:** 1 International Maize and Wheat Improvement Center (CIMMYT), Nairobi, Kenya, and; 2 International Crops Research Institute for the Semi-Arid Tropics (ICRISAT), Nairobi, Kenya

**Keywords:** Sorghum, Tanzania, Bivariate selection, Dis-adoption

## Abstract

**Purpose:**

New agricultural technologies are continuously generated and promoted for adoption by farmers with the expectation that they bring about higher benefits than older technologies. Yet, depending on the perceived benefits, the user of the technology may choose to stop using it. This paper aims to analyze what drives farmers to dis-adopt climate smart sorghum varieties in Tanzania.

**Design/methodology/approach:**

The study uses cross-sectional farm household level data collected in Tanzania from a sample of 767 households. The determinants of dis-adoption are explored using a bivariate probit with sample selection model.

**Findings:**

The authors find that while farmers switch between different sorghum varieties, most farmers actually quit sorghum production. Older farmers and those facing biotic stresses such attacks by birds are more likely to dis-adopt sorghum.

**Practical implications:**

These findings suggest that there is scope for improving and sustaining the adoption of sorghum varieties in Tanzania once extension services are strengthened. The findings also point to a well-founded theory on the role of markets in enhancing the overall sustainability of food systems.

**Social implications:**

The study findings have broader implications for understanding the sustainability of improved technology adoption

**Originality/value:**

Dis-adoption is also positively associated with the lack of access to markets underscoring the role of markets in enhancing the overall sustainability of technology adoption and food systems.

## 1 Background

### 1.1 Motivation

With over 40 per cent of the area classified as arid, food insecurity in sub Saharan Africa (SSA) is wide spread and dire. Climate change predictions for SSA suggest rainfall reduction, variable distribution pattern, increased erratic rainfall, intra-seasonal dry spells and incidences of flooding, high temperatures and higher frequency of droughts (Hadebe *et al*., 2016). There is scope for mitigating the negative impacts of climate change on food security through the development and dissemination of crops with a high ability to withstand water-stress periods. Sorghum’s drought, heat and flooding tolerance (Hadebe *et al*., 2016), and the ease of adoption by farmers makes it an ideal crop for production in SSA. *Ex ante* impact assessments have also shown that, in fact, climate change will create more favorable growing conditions for sorghum (Orr *et al*., 2016) as compared to maize. According to this study, sorghum will remain an important food crop within the SSA region, particularly in drought-prone areas where household food security cannot rely solely on maize. Sorghum’s resilience to drought will increase its importance as a source of adaptation to climate change.

In Eastern and Central Africa, sorghum is a major food security crop accounting for 41 per cent of the region’s grain production (Msongaleli *et al*., 2004). Orr *et al*. (2016) report that smallholders in Tanzania grew sorghum primarily as a food crop, responding slowly to changes in relative market prices compared to maize, but reducing the production of sorghum after a year of good rains when they had experienced a bumper harvest of maize. In addition to food and feed, it is used for a wide range of industrial purposes, including starch for fermentation and bio-energy as well as feed for livestock.

Despite its strategic importance, sorghum yields are low, averaging approximately 1000 kg ha^−1^ which has been broadly attributed to low soil fertility, bird feeding damage, striga, weed infestation, use of cultivars with low yield potentials and other socio-economic factors (Msongaleli *et al*., 2004). Resulting from increased efforts by the International Crops Research Institute for the Semi-Arid Tropics (ICRISAT) and the National Agricultural Research Institutes, several improved sorghum varieties have been released in the past four decades (Ndjeunga *et al*., 2015). While such varieties offer great promise for boosting sorghum productivity and better resistance to biotic and abiotic stresses, Schipmann *et al*. (2013) report that their adoption remains dismal and their adoption dynamics have not been fully understood. However, as expressed by Sanou *et al*. (2017), while the low technology adoption rates in the developing world may be attributed to dis-adoption (farmers who once adopted a new technology but have stopped using it), only a few studies have focused on factors affecting continuous or discontinuous use of adopted technologies, with the exception of Oladele (2005), Neill and Lee (2001) and Kim (2017).

The extent to which sorghum variety abandonment or switching is happening among sorghum farmers in Tanzania has never been fully understood. The question addressed in this study is:

In the light of the prominence of wide spread dissemination of improved sorghum varieties in Tanzania, are some varieties being adopted and then dis-adopted? If yes, what does the adoption and subsequent dis-adoption of such varieties tell us about the sustainability of sorghum production in Tanzania and the resulting lessons for sorghum breeders, policy makers and farmers?

The factors to explain adoption and dis-adoption cycles may be external, agronomic and climatological factors, or internal to the sorghum system and are the main focus of this study.

### 1.2 The sorghum sector and its importance in Tanzania

**Figure 1:**
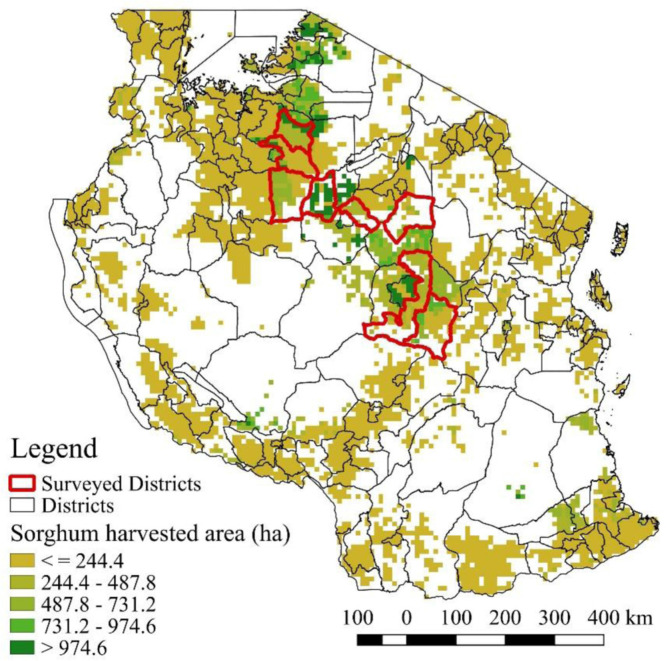
Sorghum planted area in Tanzania and surveyed districts

Tanzania is the largest producer of sorghum in Eastern and Southern Africa, occupying 21 per cent of the total cereal area in the country (Brown, 2013). It is the third most cultivated cereal after maize and rice, and it is mostly grown in the central parts (Figure 1). According to Ashimogo (1995), 90 per cent of sorghum producing households consumed the sorghum they produced.

According to the United Republic of Tanzania (2012), Singida, Shinyanga and Dodoma regions have the largest area planted to sorghum (15.5, 14.6 and 14.2 per cent, respectively) followed by Mura (12 per cent), Tabora (10 per cent) and Lindi (8 percent). The average sorghum yield for Tanzania is estimated to be approximately 1000 kg ha-1, and too low to sustain an average farm family for 12 months (Brown, 2013). Popular varieties that had been released in Tanzania prior to the data collection for this study include *Pato* (released in 1995) and *Macia* (released in 1999). Other improved varieties released in Tanzania include *Tegemeo, Wahim, Hakika, Sila, Serena and Lulu*. These new releases have been widely disseminated and are being adopted by the farming population.

Despite its low productivity, the growing demand for sorghum in Tanzania and the wider East Africa community offers a great opportunity for small-scale farmers to benefit from sorghum production. This demand has increased dramatically following a resolution by the East Africa Breweries Limited to use sorghum to produce one of its beer brands. According to figures by the Tanzanian Ministry of Agriculture, Food Security and Cooperatives, the annual demand for sorghum in Tanzania in 2011/12 was 3,360,000 metric tons, while the supply was 1,084,000 metric tons. Against the background of this highly dynamic market, both the demand and production of sorghum Tanzania are projected to increase substantially by the year 2050. The supply is projected to increase by over 300 per cent from about a million tons in 2015 to close to 5 million tons in 2050, while the demand for sorghum is expected to double from about 900 thousand tons to about two million tons by 2050, turning Tanzania into a net exporter of sorghum (Orr *et al*., 2016).

## 2 Methodology

### 2.1 Analytical framework

The decision whether to adopt improved sorghum can be modeled using the general framework of utility maximization (Uaiene *et al*., 2009; Hassen, 2015). The authors start from the assumption that farmers adopt a new technology only when the utility gained from using such a technology is significantly greater than the utility gained without using it. Even though it is not possible to observe utility directly, a farmers’ adoption decision is observed through which their utility is indirectly inferred. Following Hassen (2015), the authors let $U_{s}^{n}$ be the benefit level in the state of non-adoption (n) of improved sorghum varieties (s); $U_{s}^{a}$ the benefit level in the state of adoption (a). A farmer will transit from the state of non-adoption to the state of adoption of variety (*s*) if:

$$Y_{is}^{*}=U_{is}^{a}-U_{is}^{n}>0$$ (1)

The farmer will not adopt if:

$$Y_{is}^{*}=U_{is}^{a}-U_{is}^{n}<0$$ (2)

where $Y_{is}^{*}$ is the latent net benefit of adopting or not adopting an improved sorghum variety. As expressed by Neill and Lee (2001), the initial adoption decision and the continued use of the technology once it has been adopted can be explained by two discrete sequential decisions. Stage 1 represents the decision of whether or not to adopt sorghum, while Stage 2 represents the decision whether to dis-adopt or continue cultivating sorghum if sorghum has been adopted. The dependent variables in both stages of the decision process are dichotomous.

Given the contingent nature of the decision framework, correct and efficient estimation of determinants of dis-adoption requires a joint estimation of the two decisions and taking into account sample selection as to report dis-adoption of an improved sorghum, it is necessary to adopt it in the first place.

The adoption and abandonment transitions can be expressed in two latent equations with two latent stochastic variables $y_{1}^{*}$ and $y_{2}^{*}$ which capture the propensity to make the first and second transition in the adoption status, respectively, with the first transition representing the transition from non-adoption to adoption and second representing the transition from adoption to abandonment. Thus, the latent adoption and dis-adoption decisions are determined by:

yi2*=xi2β2+ε= 1if yi1*>0,yi2*>0 0if yi1*>0,yi2*≤0not observed yi1*≤0or yi2*≤0 (3)

Selection

$$\ln L=\sum_{i}^{N}\{y_{i1}y_{i2}(\ln\varphi_{2}(x_{1}\beta_{1},x_{1}\beta_{2,}\rho )+y_{i1}(1-y_{i2})\ln\ln\varphi_{2}(x_{1}\beta_{1},x_{2}\beta_{2},-\rho )+(1-y_{il})\ln\varphi_{1}(-x_{1}\beta_{1}\}$$ (4)

Outcome where the latent variables $y_{i1}^{*}$ and $y_{i2}^{*}$ represent the utility that the *i*_th_ household receives from adopting improved sorghum and continuing the use, respectively. They depend on vectors of farmer observed characteristics *x*_i1_ and *x*_i2_ which represent farmer observed characteristics for adopting/non-adopting sorghum and farmer observed characteristics affecting dis-continuous cultivation of sorghum, respectively; α _1_ and α _2_ are vectors of coefficients to be estimated; *μ* and *ε* are error terms.

The latent variables $y_{i1}^{*}$ and $y_{i2}^{*}$ are, by definition, unobservable but instead the authors observe two binary variables *y* indicating if individuals actually make each of the two transitions. The observable binary variables, *y*_i1_ and *y*_i2_ have the value of 1 when $y_{i1}^{*}>0$ > 0 and $y_{i2}^{*}>0$ > 0, respectively. Thus, the binary variables are defined as *y*_i1_ = 1 if a household adopts an improved sorghum variety and if *y*_i1_ =1, then *y*_i2_ could be 1 (indicating continuous adoption) or 0 (for abandonment). If *y*_i1_ = 0, then *y*_i2_ is not observed or does not exist implying that the latent variable $y_{i2}^{*}$ is only observed if $y_{i1}^{*}>0$ > 0 (Kim, 2017). In other words, it is only possible to observe $y_{i2}^{*}$ (continuation adoption decision) if a household adopted improved sorghum ($\left(y_{i1}^{*}>0\right)$ > 0). An important observation for equation %(2%) which represents continued cultivation of improved sorghum is that a smaller number of households enters the equation. This can be called the censoring of the original sample (Kim, 2017; Sanou *et al*., 2017). Indeed, as expressed by Sanou *et al*. (2017), only a subset of original sample adopts the technology [equation %(1%)], continued use is observed only for those who adopt the technology [equation %(2%)].

It is assumed that the unobserved error vector (μ ,ε ) is distributed bivariate normal with zero mean and independently to the explanatory variables *X*_i1_ and *X*_i2_ where:

$$\mu ∼N\left(0,1\right)\varepsilon ∼N\left(0,1\right)corr\left(\mu ,\varepsilon \right)=\rho$$ (5)

The log-likelihood function of the model is given by the following equation:

$$y_{i1}^{*}=x_{i1}\beta_{1}+\mu =\left\{\begin{array}{l} 1\;if\;y_{i1}^{*}\;>0\\ 0\;if\;y_{i1}^{*}\leq 0 \end{array}\right.$$ (6)

where *i* = 1,2,.. ..*N*. As expressed by Sanou *et al*. (2017) in specification (4), φ_1_ is the univariate normal distribution, and φ _2_ is the bivariate normal distribution; *y*_i1_ and *y*_i2_ are binary variables taking unity if farmer *i* adopts improved sorghum and if farmer *i* continuously uses them, respectively, and 0 otherwise. And *ρ* is the coefficient of correlation.

Our estimated conditional probability derived from equations %(1%) and %(2%) is a bivariate Probit with sample selection model (Heckman, 1976)[1]. A bivariate probit model allows for a continuous structure of utility between the two decisions. It provides a correlation term *ρ* that represents how the unobserved characteristics affecting utility maximization, implicit in the first decision, are related to the second (Neill and Lee, 2001). If the null hypothesis that *ρ* = 0 cannot be rejected, there is no correlation between the error terms of the two equations, and they may be estimated with separate probit specifications.

### 2.2 Data

The data for the study are based on a survey of 767 households from 57 villages across 8 of the major sorghum growing districts in mainland Tanzania and collected by ICRISAT in collaboration with Selian Crops Research Institute of Tanzania in 2011. The villages for the survey were drawn from the National Master Sample (NMS) developed by the National Bureau of Statistics (NBS) to serve as a national framework for conducting household-based surveys in Tanzania developed from the 2002 population and housing Census. A multistage sampling procedure was used in the selection of households for the study. The first stage involved the selection of major sorghum growing regions. The second stage involved the selection of major sorghum growing districts followed by the selection of villages and the households from each of the selected villages.

Four major sorghum growing regions (Dodonoma, Shinyanga, Singida and Tabora) were selected for the survey. The locations of the eight districts within these four regions where the study was conducted are highlighted in Figure 1.

The sampling process was proportional to the size with 5-8 villages selected per district leading to the total of 60 villages. In total, 15-30 households were randomly sampled per village based on population of the village, leading to the sampling of 800 households.

### 2.3 Model specification

The model specification involves assigning *y*_1_ =1 in equation %(1%) above for the first adoption decision and assigning *y*_2_ = 1 for the continuous adoption. Non-adoption is represented by *y*_1_ = 0 and abandonment of the technology by *y*_2_ = 0. As expressed by Neill and Lee (2001), this specification implies that positive coefficients in both decisions are associated increasing the probability of growing improved sorghum varieties, while negative coefficients will be associated with a decreasing probability. The two separate equation specifications are estimated taking into account a number of variables such as farm size, market access, incidence of biotic stresses as well as other socio-economic variables. Table III depicts expected coefficient signs for the variables included in both equations.

#### 2.3.1 Dependent variables

The dependent variables for the study relate to adoption and the subsequent dis-adoption. The analysis is based on two interrelated dependent variables. One reflects whether a farm household has ever cultivated at least one improved sorghum variety. The other dependent variable reflects dis-adoption patterns, where dis-adoption is defined as ever-growing an improved variety before 2011, but did not grow it in the year of survey in 2011 for whatever reason. The authors ran one pooled regression involving all improved varieties and four separate regressions for each of the improved varieties (*serena, macia, tegemeo and pato*).

#### 2.3.2 Description of explanatory variables and hypotheses

The variables that are hypothesized to influence adoption and dis-adoption of improved sorghum varieties were selected based on a review of theoretical work and previous empirical adoptions studies (Feder *et al*., 1985; Diagne, 2006; Kassie *et al*., 2013). Below, a brief description of the variables and a priori expectation on their effect on adoption and dis-adoption is presented (Table I).

**Table I:** Description of some key explanatory variables and expected signs

		Expected signs	
Explanatory variables	Definition	Adoption	Continued adoption
*Household socio-demographics*			
Gender	Equals 1 if household head is male and 0 otherwise	+/-	+/-
Education	Years of education of head of household	+	+
Age	Age of the household head in years	+/-	+/-
Household size	Number of people living in one household	-	-
Farm size	Number of hectares owned by household	+	+
Livestock units	Number of livestock units owned	+	+
Off Farm	Equal 1 if farmers expressed participation in off farm self-employment: 0 otherwise	+/-	+/-
*Exposure and social capital*			
Source of extension information	Equal 1 if government is source of extension (base) compared against other extension service providers (Farmer club, NGO, Research Centre., Seed/grain dealer, Another farmer)	-	-
Neighbors	Number of friends and neighbors the farmer saw growing improved sorghum	+/-	+/-
*Community characteristics*			
Distance to the market	Distance to the nearest market in km		
Disease and pest	Equal 1 if farmers expressed existence of disease and pest problem; 0 otherwise	+/-	+/-
Seed constraint	Equal 1 if farmers in the village expressed existence of seed constraint: 0 otherwise	-	-
Drought	Equal 1 if farmers in the village expressed existence of drought problem: 0 otherwise	-	-

##### 2.3.2.1 Household demographic and socioeconomic characteristics

Empirical studies (Adesina and Baidu-Forson, 1995; Uaiene *et al*., 2009) have shown that the age of a household’s head, which captures his or her farming experience could influence adoption decision, either positively or negatively. Adesina and Baidu-Forson (1995) finds that age positively influenced the adoption of sorghum in Burkina Faso, while Polson and Spencer (1991) observe the contrary when they find that the younger farmers are more risk-taking and willing to uptake an improved technology. This makes it difficult to predict the impact of age on the continuity of the technology adoption. It is more difficult to predict the effect of gender on sorghum technology adoption and dis-adoption, although Schipmann *et al*. (2013) showed no gender impacts on sorghum adoption. The size of the household, is a proxy for the availability of labour. Generally, the production of improved sorghum varieties is relatively less labour intensive as Schipmann *et al*. (2013) report. Thus, the authors expect the coefficient to have a negative sign. The size of the land owned can have an impact of whether or not to adopt improved varieties. Generally, varieties that produce higher yields are likely to be attractive to those with small land holdings, leading to an expected negative sign for the coefficient for both the adoption and the abandonment equations. However, land is also a wealth proxy variable which can have a positive effect on the adoption of improved sorghum varieties (Feder *et al*., 1985).

The participation in off-farm self-employment can have unpredictable impacts on adoption and dis-adoption. Reasons for participation in off-farm employment include:

- self-insurance against risk;
- an ex-post coping strategy;
- inability to specialize due to incomplete input markets; and
- consumption diversification where there are incomplete output markets.

Thus, it is hard to predict the sign of the coefficient on both adoption and dis-adoption.

##### 2.3.2.2 Exposure and social capital variables

Exposure and social capital variables are crucial drivers of adoption decisions of any technology. The knowledge of any friends and neighbors that grow improved sorghum is critical in adoption decisions as it exposes farmers to a new technology and increases the probability of adoption as reported in other studies (Diagne, 2006). However, a prediction on its impact on abandonment is not possible. As expressed by Schipmann *et al*. (2013), the information source for new sorghum cultivars plays an important role in adoption.

##### 2.3.2.3 Community variables, biotic and abiotic factors

Market access was assessed using the distance to the main market which reflects transaction costs associated with buying inputs and taking produce to the market. Apart from affecting the access to the market, these distances can also affect the availability of new technologies, information and credit institutions (Kassie *et al*., 2013). The authors therefore expect the relationship between the distance to the market and adoption of improved sorghum varieties to be negative for both, the first adoption equation and the continued adoption equation as the incentive to produce sorghum is expected to be lower the further away from the market. Moreover, the authors also want to understand the effect of abiotic and biotic stresses such as drought, pest and diseases on the adoption as well as the sustainability of technology adoption. The occurrence of drought is likely to make sorghum production attractive and more so if improved varieties are drought tolerant or if they have short growth cycles favorable for the semi-arid areas, while the occurrence of pests and diseases and the lack of seed will have a negative effect on the adoption and encourages variety abandonment.

## 3 Results

### 3.1 Descriptive results

#### 3.1.1 Types of sorghum varieties planted

Table II presents sorghum varieties grown in Tanzania and their rates of abandonment. The levels of adoption for different sorghum varieties vary extensively across farmers. Column 3 shows the numbers of farmers expressing that they ever grew the variety of sorghum. Local sorghum varieties are quite popular in Tanzania with all farmers reporting to have ever grown local sorghum varieties and 76 per cent reporting growing them in the year of the survey. Among improved varieties, *Macia, Serena, Pato* and *Tegemeo* were the most popular varieties, a finding consistent with Schipmann *et al*. (2013). *Serena, Pato* and *Macia* are widely preferred for their tolerance to striga (*Striga hermonthica, Striga asiatica, and Striga forbesii*), while *Tegemeo* is highly susceptible to striga. Column 5 depicts the rate at which farmers are abandoning each variety regardless of the time when the varieties were abandoned. *Tegemeo, Pato* and *Serena* varieties recorded the highest abandonment rates of 58.4, 43.8 and 41.6 per cent by the farmers that had ever grown the varieties, respectively. While *Macia* was the most widely cultivated variety, only 22.8 per cent of the sorghum farmers did not grow the variety during the year of the survey. The low dis-adoption rate of *Macia* relative to other improved varieties may suggest that the *Macia* has some preferable characteristics that encourage farmers to continue growing the variety, but it could as well be that the variety is relatively new compared to the others.

**Table II:** Types of sorghum varieties planted and dis-adopted

Sorghum variety	Year of release	Ever planted (%) (*n* = 767)	Planted in 2011 (%) (*n* = 767)	(%) dis-adoption
Local sorghum	–	100.0	75.9	24.1
*Serena*	1960	10.0	5.9	41.6
*Pato*	1977	14.6	8.2	43.8
*Tegemeo*	1978	11.6	4.8	58.4
*Macia*	1998	30.2	23.3	22.8
*Wahi*	2002	5.1	4.0	20.5
*Hakika*	2002	0.8	0.5	33.3
*Sila*	2008	0.5	0.3	50.0

**Table III:** Transitions in variety cultivation (%)

	Variety switched to					
Variety dis-adopted	Serena	Pato	Tegemeo	Macia	Local	Quit sorghum
*Serena* (*n* = 32)	0.0	3.1	3.1	6.3	21.9	65.6
*Pato* (*n* = 52)	3.8	0.0	0.0	17.3	48.1	30.8
*Tegemeo* (*n* = 49)	2.0	0.0	0.0	28.6	36.7	32.7
*Macia* (*n* = 53)	0.0	7.5	1.9	0.0	32.1	58.5

**Figure 2:**
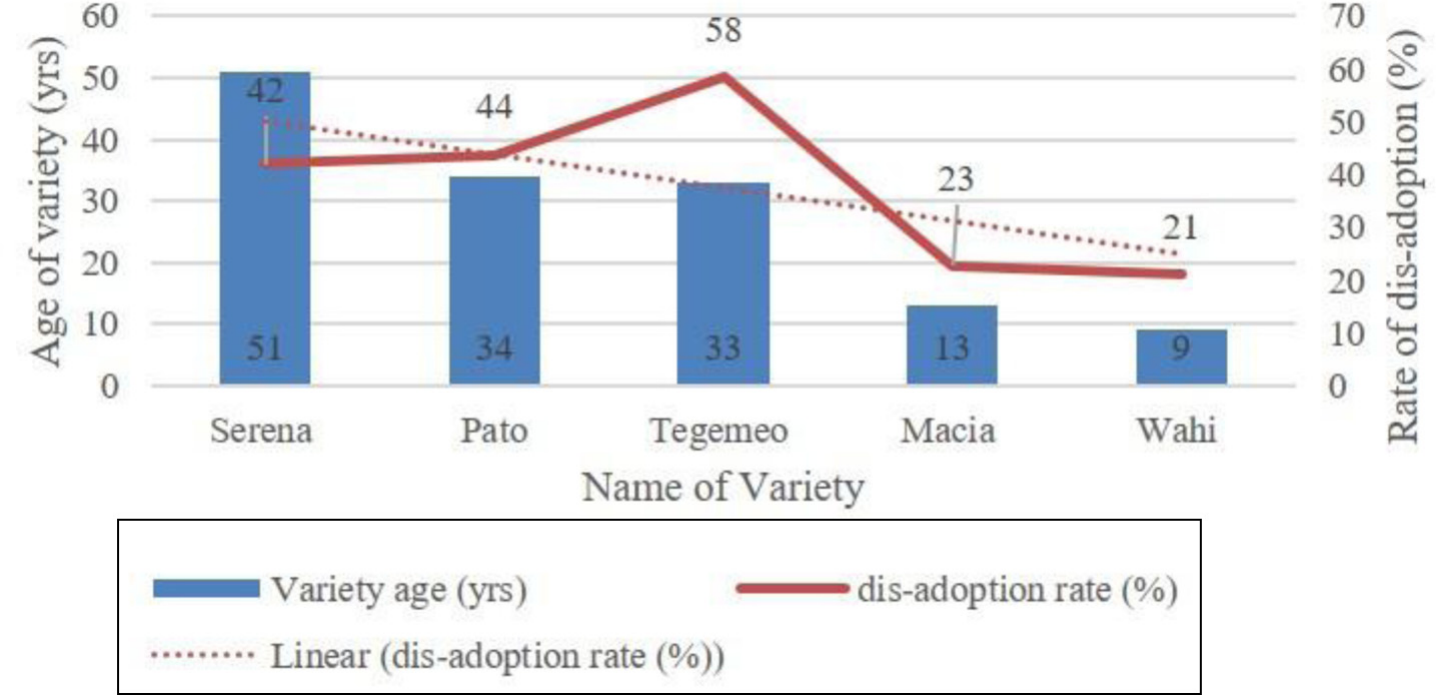
Variety age versus rate of disadoption

Moreover, older varieties may also be suffering from the lack of seed. It is likely that sorghum seed companies are focusing on new improved sorghum varieties than the older varieties, making it difficult for farmers to access improved seed for older varieties such as *Serena, Tegemeo* and *Pato*. As depicted in Figure 2, there is a correlation between the age of the variety and the rate of its abandonment with older varieties such as *Serena* (50 yrs), *Pato* (34 yrs) and *Tegemeo* (33 yrs) registering higher dis-adoption rates of 42, 44 and 58 per cent, respectively.

#### 3.1.2 Transitions in variety cultivation

As expressed by Westengen and Brysting (2014), switching to other varieties within the farming communities is an important adaptation strategy within a diverse portfolio of livelihood responses to multiple stresses. Table III depicts results of the adoption dynamics among sorghum farmers. Most farmers that had abandoned *Serena*, actually did not grow any sorghum variety (65.7 per cent), while a few (21.9 per cent) switched to the cultivation of mostly local varieties. Consistent with dis-adopters of *Serena*, most farmers that dis-adopted *Macia* (58.5 per cent) also did not grow any sorghum variety while 32.1 per cent of them switched to local varieties. A significant proportion of previous growers of *Tegemeo* and *Pato* also either quit sorghum production or switched to the cultivation of local sorghum varieties. Variety switching within improved varieties is largely limited to *Macia*, with 17.3 per cent and 28.6 per cent of the previous growers of *Tegemeo* and *Pato*, respectively, switching to *Macia.* The findings largely suggest that most sorghum farmers are moving out of sorghum cultivation to other enterprises.

#### 3.1.3 Reasons for dis-adopting of sorghum varieties

The qualitative information on what motivates the dis-adoption of certain varieties of sorghum is depicted in Table IV. This information is quite useful, as it allows for a much richer analysis of farmer’s decision-making and underlying rationale with regards to technologies (Grabowski *et al*., 2016). It allows for a greater contextualization of the econometric analysis that follows later. Overall, the lack of seed is featured highly as the main reason for abandoning sorghum cultivation, but the problem is more pronounced among those who had planted *Pato* in the past. This underscores the need to strengthen sorghum seed systems as a way of sustaining sorghum production. Local varieties were abandoned due to their susceptibility to drought and low yields. Attacks by diseases and pests on *Macia* and birds on *Tegemeo* and the lack of market for *Serena* sorghum varieties were among the most frequently reported reasons for abandoning the cultivation of some sorghum varieties.

**Table IV:** Reasons for disadopting the sorghum production (%)

Reason for dis-adopting	Local (*n* = 165)	Serena (*n* = 32)	Tegemeo (*n = 52*	Pato (*n* = 49)	Macia (*n* = 53)	Total (*n* = 351)
Lack of seed	10.9	19.3	34.5	57.2	30.2	25.3
Requires more rainfall	47.3	5.3	6.9	1.8	1.9	21.7
Diseases and pests	10.3	14.0	13.8	10.7	34.0	14.5
Birds attack	2.4	15.8	27.6	17.9	20.8	16.2
Lack of market and poor prices	12.1	33.5	12.1	3.6	7.5	9.4
Low yielding variety	11.5	1.8	0.0	0.0	5.7	5.7

#### 3.1.4 Socioeconomic and demographic characteristics by adoption status

Descriptive statistics for selected variables for the sampled households disaggregated by the adoption status of improved sorghum varieties are presented in Table V. About three in four farmers were male, while the average age was 44 years among the sampled households. An average household was comprised of about six members with an average land holding size of four hectares. About 70 per cent of the respondents expressed knowledge of friends/neighbors that grew improved sorghum varieties. A significantly higher proportion of adopters (92 per cent) knew of some farmer/friend growing improved sorghum varieties compared to only 48 per cent for the non-adopters. The average distance to the main markets was 22 kilometers, and adopters were closer to the market (23.9 km) than those that abandoned (30 km) the cultivation of improved varieties. In terms of sources of extension information, the majority (59 per cent) expressed accessing information through other farmers, while 35 per cent of the farmers accessed information through government extension workers. A significantly higher proportion of non-adopters (77 per cent) reported access extension information through other farmers as compared to adopters (45 per cent) a finding that suggests that over-reliance on informal extension systems may be ineffective in the diffusion of appropriate and improved technologies among farmers. On the contrary, only 17 per cent of non-adopters accessed information through a conventional government extension system compared to 48 per cent for the adopters and 57 per cent for the abandoners.

**Table V:** Means statistics for adopters, abandoners and no adopters

	Adopters (*n* = 289)	Abandoners (*n* = 119)	Non-adopters *(n* = 359)	Total (*n* = 767)
Gender of household head (1 = male, 0 =female)	0.74	0.74	0.77	0.76
Age of the household member (yrs)	43.15	45.22	45.18	44.42
Years of education (yrs)	2.85	2.88	2.82	2.84
Household size	5.92	5.89	6.03	5.97
Distance to the main market (km)	23.9	30.0	18.1	22.1
Land holding size (ha)	4.87	3.43	3.95	4.21
Livestock units (LU)	6.4604	8.1718	6.8375	6.903
Whether knows friends growing sorghum (yes = 1, 0 = otherwise)	0.92	0.85	0.48	0.71
Number of friends known planting sorghum	15.58	17.36	5.60	11.19
Years of experience in sorghum farming (yrs)	13.88	13.46	13.86	13.8
*Sources of information*				
Government (yes = 1, 0 = otherwise)	0.48	0.57	0.17	0.35
Farmer club (yes = 1, 0 = otherwise)	0.01	0.01	0.03	0.02
NGO (yes = 1, 0 = otherwise)	0.01	0.02	0.01	0.01
Research Centre (yes = 1, 0 = otherwise)	0.02	0.03	0.01	0.02
Seed/grain dealer (yes = 1, 0 = otherwise)	0.01	0.02	0.01	0.01
Another farmer (yes = 1, 0 = otherwise)	0.45	0.36	0.77	0.59
Participation in off farm employment (yes =1, 0 = otherwise)	0.22	0.26	0.26	0.25

### 3.2 Econometric results

#### 3.2.1 Determinants of adoption of all improved varieties

The determinants of sustained adoption for all improved sorghum varieties are presented in Table VI based on the maximum likelihood estimates from a Heckman bivariate probit. The estimation was done in three models: Model 1 has no extension services and membership in social grouping variables, Model 2 includes extension services, while Model 3 includes membership in social groupings. The inclusion of the extension services in Model 2 slightly alters some results, but does not change the coefficients much compared to Model 1, suggesting that the district dummies sufficiently control for the observable factors. The model chi-square, which measures the goodness of fit of the model is significant at 1 per cent level, indicating a good fit. The correlation term, *ρ* is not significant suggesting that the unobservable attributes that affect the decision to adopt improved sorghum varieties do not affect the decision to continue growing these varieties. Although the results suggest that the residuals of the two probit equations are not significantly correlated, the maximum likelihood results are maintained as simultaneous estimation by maximum likelihood is more efficient than separate estimation of each of the probit equations. The age of the head of household does not significantly influence the first adoption of improved sorghum varieties, but the relationship between age and the first adoption status is nonlinear as the square of age has a positive and significant effect on the first adoption suggesting that as farmers grow beyond some tipping point of age.

**Table VI:** Bivariate heckman probit estimates of the adoption and abandonment of improved sorghum varieties

	Model (1)		Model (2)		Model (3)	
Explanatory variables	Adoption	Cont.use	Adoption	Cont.use	Adoption	Cont.use
Gender (1 = male,0 = female)	−0.12 (0.13)	0.06 (0.18)	−0.04 (0.14)	0.01 (0.19)	−0.090.14	0.01 (0.19)
Age of household head yrs)	−0.38 (0.38)	−18.69 (11.98)	−0.62 (0.38)	−20.51* (11.73)	−0.69* (0.39)	20.27*** (7.71)
The square of age	0.01* (0.00)	2.45 (1.57)	0.01* (0.00)	2.69* (1.55)	0.00** (0.0.00)	2.67*** (1.02)
Education (0 = low, 1 = high)	0.19 (0.17)	−0.02 (0.25)	0.13 (0.17)	0.03 (0.26)	0.10 (0.17)	0.02 (0.26)
Household size	0.04 (0.15)	0.12 (0.25)	0.05 (0.16)	0.16 (0.26)	0.08 (0.15)	0.20 (0.22)
Distance to market (km)	0.01 (0.03)	0.10*** (0.04)	0.02 (0.03)	−0.11*** (0.04)	0.04 (0.03)	0.11*** (0.04)
Land holding size (ha)	0.02 (0.04)	0.08 (0.07)	0.02 (0.04)	0.09 (0.06)	0.03 (0.03)	0.07 (0.06)
Total livestock units	0.05** (0.03)	−0.03 (0.04)	0.03 (0.03)	0.00 (0.04)	0.03 (0.03)	−0.01 (0.04)
Quality of house roof (1- = iron sheet, 0 = otherwise)	0.45*** (0.15)	0.15 (0.19)	0.42*** (0.15)	0.11 (0.19)	−0.46*** (0.15)	0.13 (0.20)
Quality of wall (1 = bricks, 0 otherwise)	0.04 (0.19)	0.28 (0.30)	−0.03 (0.19)	0.26 (0..31	−0.02 (0.19)	0.24 (0.31)
Neighbors growing improved sorghum	0.21*** (0.02)	−0.07 (0.13)	0.19*** (0.02)	−0.03 (0.14)	0.20*** (0.02)	−0.03 (0.05)
Participation in off farm employment (1 = yes, 0 =0therwise)	0.09 (0.18)	−0.10 (0.26)	0.04 (0.15)	−0.12 (0.23)	0.08 (0.14)	−0.16 (0.22)
Seed constraints (1 = yes, 0 = 0therwise)	0.29 (0.23)	−2.11*** (0.43)	0.22 (0.25)	−2.16*** (0.43)	0.26 (0.26)	−2.13*** (0.44)
Disease and pests (1 = yes, 0 = 0therwise)	−0.07 (0.27)	−1.79*** (0.4)	−0.30 (0.27)	−1.81*** (0.41)	−0.33 (0.26)	−1.77***0.43)
Birds constraint (1 = yes, 0 = 0therwise)	0.95**(0.45)	−6.67*** (0.99)	0.78 (0.48)	−7.69*** (0.42)	0.80 (0.50)	−8.08*** (1.23)
*Main sources of information (base-government extension agents)*						
Farmer club members			−1.02*** (0.33)	0.07 (0.47)	−0.99*** (0.32)	0.04 (0.59)
Research Centre			0.13 (0.42)	0.33 (0.42)	0.08 (0.37)	0.03 (0.51)
Seed stockiest			0.11 (0.49)	0.61 (0.75)	0.16 (0.51)	0.52 (0.73)
Another farmer			−0.72*** (0.13)	−0.12 (0.23)	−0.74*** (0.13)	0.35 (0.22)
Membership in farmers association (1=member; 0 = otherwise)					0.41** (0.18)	0.05 (0.23)
District dummies	Yes	Yes	Yes	Yes	Yes	Yes
Constant	0.46 (1.56)	39.2* (20.7)	0.18 (1.14)	36.6 (21.4)	1.26 (1.14)	38.81***14.14
*ρ*	0.70 (0.52)		0.461.57)		−0.54 (0.36)	
Observations	766		766		766	
Censored obs	359		359		359	
Wald chi2	198.5***		394.5***		284.6***	

Notes: *, **, ***represent significance at 10, 5 and 1%, respectively; Figures in parentheses are standard errors

The effect of extension services was assessed by including different sources of extension information in the regression as dummy variables and using government extension as a reference point. Highlighting the importance of government extension systems, farmers that relied more on members of farmer club and fellow farmers in accessing agricultural information were less likely to adopt improved sorghum compared to farmers that accessed most of the information through the government extension system. The coefficient for research organization as sources of information was positive but not significant suggesting that accessing information through research organizations did not significantly influence the adoption of improved sorghum varieties. Indeed, the research organizations’ lack of positive influence on farmer’s adoption could indicate a more important underlying problem of failure to communicate effectively with farmers which should be carefully looked into from the research organizations’ perspective.

Underscoring the role of access to appropriate social networks, the coefficient for the number of neighbours known by the farmer that grow improved sorghum varieties was positive and significant at 1 per cent level suggesting that farmers with proximity to neighbours growing improved sorghum varieties increased the propensity for adoption. Furthermore, consistent with prior expectation and the observed influence of others farmers as information sources, membership in a farmer’s association significantly and positively affects the household’s adoption decision. The positive relationship could be attributed to positive peer effects in sorghum adoption. However, as expressed by Orr *et al*. (2016) sorghum has a reputation as a “poor man’s crop” for which demand declines as income rises as such the results suggest that well-off farmers are less likely to grow improved sorghum. The negative effect of the ownership of residential houses that are roofed with iron sheets as opposed to a grass thatch on the adoption of improved sorghum varieties is consistent with this notion of sorghum being mainly grown by the poorer households.

#### 3.2.2 Determinants of continued use of improve sorghum varieties

The conditional decision to abandon or sustain sorghum cultivation shows that the age of the farmers is a significant determinant of whether a farmer sustains the cultivation of improved sorghum over a longer period. The age of a farmer was negatively associated with continued sorghum cultivation perhaps reflecting an increase in the viability of non-sorghum enterprises over time. However, signifying the non-linear relationship between age and continued cultivation, the squared of age was positive indicating an increasing negative effect on continued adoption. The coefficient for the distance to the input and output market was negative and significant (at 1 per cent level) suggesting that households far from the market were more likely to abandon the cultivation of improved sorghum varieties than those close to markets. The results are consistent with the well-established theory around the positive role of markets in propelling the sustainability of food systems. Through access to markets, farmers may access inputs while also finding the opportunity for marketing their products. The importance of market access could also be based on the possibility that modern varieties may not always be consistent with the households’ consumption demands but are tailored to other markets like the brewing industry. In fact, most of the sorghum varieties released in Tanzania are recommended for beer processing. With regards to this, Orr *et al*. (2016) report that in Tanzania, the use of sorghum for food processing exceeds the use of sorghum for food. Most of the abiotic and biotic constraints were found to be responsible for the abandonment of sorghum cultivation. The bird problem is endemic to Tanzanian sorghum farmers who feel researchers are not doing enough to address the problem. Consistent with this notion Laswai *et al*. (2008) report that the bird problem has been neglected. Breeders, who have succeeded in improving the yield of compact-headed sorghums that are more easily attacked by birds do not seem to see the damage caused as a problem needing immediate attention. Accordingly, some new varieties such as *Tegemeo* and *Serena* remain highly susceptible to birds with farmers growing *Serena* sometimes reporting crop losses of up to 100 per cent as a result of bird damage (Monyo *et al*., 2004). Sorghum varieties with loose heads, like the local varieties, could offer a solution to the bird problem and make these attractive to many farmers.

#### 3.2.3 Determinants of abandonment for specific improved sorghum varieties

Table VII presents results of dis-adoption of the four most widely cultivated improved sorghum varieties: *Macia, Pato, Serena* and *Tegemeo*. The results on variety abandonment show that the age of the farmer has a negative influence on the continued adoption of *Macia*, suggesting that conditional on first adoption, younger farmers abandon the cultivation of *Macia.* The abandonment of *Macia* could be attributed to the fact that it is more susceptible to kernel smut than either *Tegemeo* or *Pato* (Monyo *et al*., 2004). Age does not significantly influence the continued adoption of *Serena, Tegemeo* and *Pato.* The coefficient for the size of the household in the *Macia* regression was positive and significant suggesting that larger households were more likely to continue cultivating *Macia* over a long period. The distance to the market was negative was only significant for two varieties (*Tegemeo* and *Macia*) which suggests that households far away from the market tend to abandon the cultivation of these two varieties if they have adopted them before.

**Table VII:** Bivariate heckman probit estimates of adoption and abandonment of specific improved sorghum varieties

	Serena				Tegemeo				Pato				Macia			
Variables	Continued adoption		First adoption		Continued adoption		First adoption		Continued adoption		First adoption		Continued adoption		First adoption	
	Coeff.	Se	Coeff.	Se	−0.266	0.420	0.146	0.150	Coeff.	Se	Coeff.	Se	Coeff.	Se	Coeff.	Se
Gender	−0.119	0.307	0.277*	0.164	−0.677	0.474	0.125	0.154	0.132	0.282	0.161	0.153	0.149	0.243	−0.031	0.146
Age	8.177	9.033	−0.526	0.413	−22.370	16.951	−0.062	0.481	−5.422	14.035	0.079	0.417	−36.338***	12.502	−0.283	0.439
Age squared	−1.051	1.193	0.000	0.000	2.860	2.248	−0.000	0.000	0.644	1.842	0.000	0.000	4.844***	1.679	0.000	0.000
Education	−0.196	0.246	0.151	0.208	−0.234	0.570	0.188	0.224	0.159	0.400	0.085	0.190	0.355	0.416	0.229	0.177
Household size	−0.076	0.278	−0.40**	0.175	−0.188	0.547	0.040	0.151	0.410	0.349	0.084	0.162	0.794***	0.303	0.243	0.151
Distance to market	0.053	0.031	0.001	0.018	−0.198*	0.107	0.076**	0.034	−0.016	0.061	0.054*	0.028	−0.246***	0.084	0.021	0.029
Land size	0.062	0.061	−0.021	0.032	0.288**	0.119	−0.016	0.036	0.063	0.104	0.069*	0.036	0.087	0.082	−0.011	0.042
Livestock units	0.039	0.040	0.067*	0.035	0.003	0.091	0.014	0.030	0.056	0.050	0.007	0.028	−0.055	0.059	0.040	0.029
Neighbors growing			0.04**	0.020			0.122***	0.025			0.158***	0.024	0.149	0.243	0.168***	0.027
improved sorghum Off farm	0.004	0.247	−0.198	0.174	−0.412	0.504	0.415***	0.154	−0.112	0.270	0.008	0.008	−0.188	0.271	0.051	0.157
Employment Farmer club members			0.200	0.373			0.450	0.597			−0.489	0.520			−0.645	0.489
*Main sources of information (base-government extension agents)*																
Non-governmental			0.342	0.443			0.705	0.619			0.437	0.528			−4.950***	0.229
Org Research			−0.9***	0.258			0.284	0.453			0.857**	0.365			−0.009	0.475
Organizations Seed/grain stockiest			0.782**	0.367			−1.202**	0.567			0.838*	0.484			−0.124	0.784
Another farmer			−0.5***	0.139			−0.313**	0.159			−0.035	0.130			−0.429***	0.137
Bird constraints					−6.4**	2.58	−1.39**	0.54			0.838*	0.484			−0.124	0.784
Pest and disease					−0.835	0.742	−0.662***	0.227			−0.035	0.130			−0.429***	0.137
constraint																
Drought constraint									−0.552	0.583	−0.194	0.296				
Constant	−17.161	16.862	0.460	1.307	46.439	31.472	−1.438	1.482	9.857	26.784	−2.198*	1.311	65.136***	23.248	−1.257	1.351
Athrho		12.404***	0.095			−0.247	1.039			0.952*	0.576			0.618	0.591	
Observations	766				766				766				766			
N_censored	689				677				654				535			

## 4 Conclusions

This paper analyzes the factors affecting the adoption and abandonment of sorghum varieties in Tanzania using the bivariate selection model. The results indicate that sustained cultivation of improved sorghum varieties highly depends on the extent of access to output and input markets. Through access to markets, farmers may access inputs such as seed, while also finding the opportunity for marketing their products. The lack of access to seed was reported as a major reason for abandoning sorghum, and this can be a big constraint where farmers are far from seed markets. The significance of market access in sustaining the cultivation of all sorghum varieties underscores the need to address market failure among farmers. Moreover, young farmers are less likely to abandon the cultivation of improved varieties which clearly indicates an avenue for extension to specifically focus on this group and makes it likely that Sorghum will play a more significant role in the future. The occurrence of biotic stresses such as diseases and birds, however, significantly influences farmers to abandon the cultivation of improved sorghum varieties. The bird problem mainly encourages the abandonment of the *Tegemeo* variety but clearly highlights the need for this issue to be taken serious in future breeding efforts.

These findings suggest that there is hope for improving and sustaining the adoption of sorghum varieties in Tanzania once extension services are strengthened and breeding programs also focus on farmers needs and not purely on industry demands. Accordingly, for some varieties such as *Tegemeo*, minimizing bird and disease related losses should be considered a priority if abandonment by farmers is to be reduced and sorghum production to be sustained.

## References

[cit0001] Adesina A.A. and Baidu-Forson J. (1995), “Farmers’ perceptions and adoption of new agricultural technology: evidence from analysis in Burkina Faso and Guinea, West Africa”, Agricultural Economics, Vol. 13 No. 1, pp. 1-9.

[cit0002] Ashimogo G.C. (1995), “Peasant Grain Storage and Marketing in Tanzania: A Case Study of Maize in Sumbawanga District”, PhD thesis, University of Berlin, Verlag Koester, Berlin.

[cit0003] Brown D. (2013), “Contribution of sorghum production towards household food security in Tanzania: A Case Study of Singida region”, Thesis submitted to Sokoine University, Morogoro.

[cit0004] Diagne A. (2006), “Diffusion and adoption of NERICA rice varieties in côte d’Ivoire”, The Developing Economies, Vol. 44 No. 2, pp. 208-231.

[cit0005] Feder G., Just R.E. and Zilberman D. (1985), “Adoption of agricultural innovations in developing countries: a survey”, Economic Development and Cultural Change, Vol. 33 No. 2, pp. 255-297.

[cit0006] Grabowski P.P., Kerr J.M., Haggblade S. and Kabwe S. (2016), “Determinants of adoption and disadoption of minimum tillage by cotton farmers in Eastern Zambia”, Agriculture, Ecosystems and Environment, Vol. 231, pp. 54-67.

[cit0007] HarvestChoice (2015), Sorghum Harvested Area (ha, 2005), International Food Policy Research Institute, Washington, DC, and University of MN, St. Paul, MN, available at: http://harvestchoice.org/data/sorg_h

[cit0008] Hadebe S.T., Modi A.T. and Mabhaudhi T. (2016), “Drought tolerance and water use of cereal crops: a focus on sorghum as a food security crop in Sub-Saharan Africa”, Journal of Agronomy and Crop Science, Vol. 203 No. 3, pp. 177-191.

[cit0009] Hassen S. (2015), “Disadoption, substitutability, and complementarity of agricultural technologies: a random effects multivariate probit analysis”, Environment for Development Discussion Paper Series, EfD DP 15-26, Washington, DC, available at: www.rff.org/files/document/file/EfD-DP-15-26.pdf

[cit0010] Heckman J. (1976), “The common structure of statistical models of truncation, sample selection and limited dependent variables and a simple estimator for such models”, Annals of Economic and Social Measurement, Vol. 5, pp. 475-492.

[cit0011] Kassie M., Jaleta M., Shiferaw B., Mmbando F. and Mekuria M. (2013), “Adoption of interrelated sustainable agricultural practices in smallholder systems: evidence from rural Tanzania”, Technological Forecasting and Social Change, Vol. 80 No. 3*, pp.* 525-540*.*

[cit0012] Kim J. (2017), “Adoption and continued use of hybrid rice: Case of Haryana State, India”, Master thesis submitted to Lund University, Lund, available at: http://lup.lub.lu.se/luur/download?func=downloadFile&recordOId=8919343&fileOId=8919344

[cit0013] Laswai H.S., Shayo L.N.B. and Kundi S.T.P. (2008), “Collaborative project to investigate consumer preferences for selected sorghum and millet products in the SADC region of Africa”, AFRIPRO Workshop Proceedings, Pretoria, available at: www.afripro.org.uk/papers/paper07laswai.pdf

[cit0014] Monyo E.S., Ngereza J., Mgonja M.A., Rohrbach D.D., Saadan H.M. and Ngowi P. (2004), “Adoption of improved sorghum and pearl millet technologies in Tanzania”, Report, International Crops Research Institute for the Semi-Arid Tropics (ICRISAT) Zimbabwe, Bulawayo.

[cit0015] Msongaleli B., Rwehumbiza F., Tumbo S.D. and Kihupi N. (2004), “Sorghum yield response to changing climatic conditions in semi-arid Central Tanzania: evaluating crop simulation model applicability”, Agricultural Sciences, Vol. 05 No. 10, pp. 822-833.

[cit0016] Ndjeunga J., Mausch K. and Simtowe F. (2015), “Assessing the effectiveness of agricultural R&D for groundnut, pearl millet, pigeonpea, and sorghum in west and Central africa and east and Southern africa’, chapter 7”, in Walker T. and Alwang J. (Eds), Crop Improvement, Adoption, and Impact of Improved Varieties in Food Crops in Sub-Saharan Africa, CAB international, Wallingford, pp. 123-147.

[cit0017] Neill S.P. and Lee D.R. (2001), “Explaining the adoption and disadoption of sustainable agriculture: the case of cover crops in Northern Honduras”, Economic Development and Cultural Change, Vol. 49 No. 4, pp. 793-820.

[cit0018] Oladele 0. (2005), “A tobit analysis of propensity to discontinue adoption of agricultural technology among farmers in southwestern Nigeria”, Journal of Central European Agriculture, Vol. 6 No. 3, pp. 249-254.

[cit0019] Orr A., Mwema C., Gierend A. and Nedumara S. (2016), “Sorghum and millets in Eastern and Southern africa. Facts, trends and outlook”, ICRISAT Research Program, Markets, Institutions and Policies Working Paper Series No. 62, International Crops Research Institute for the Semi- Arid Tropics, Patancheru.

[cit0020] Polson R.A. and Spencer D.S. (1991), “The technology adoption process in subsistence agriculture: the case of cassava in southwestern Nigeria”, Agricultural Systems, Vol. 36 No. 1, pp. 65-78.

[cit0021] Sanou B., Savadogo K. and Sakurai T. (2017), “Determinants of adoption and continuous used of improved maize seed in Burkina Faso”, Japanese Journal of Agricultural Economics, Vol. 19 No. 0, pp. 21-26.

[cit0022] Schipmann C., Orr A., Muange E. and Mafuru J. (2013), “Harnessing opportunities for productivity enhancement for sorghum and millets (HOPE)”, ICRISAT Socioeconomics Discussion Paper Series, Number 7, International Crops Research Institute for the Semi-Arid Tropics (ICRISAT), Nairobi.

[cit0023] Uaiene R.N., Arndt C. and Masters W.A. (2009), “Determinants of agricultural technology adoption in Mozambique”, Discussion Papers, 67E, National Directorate of Studies and Policy Analysis, Ministry of Planning and Development.

[cit0024] United Republic of Tanzania (2012), National Sample Census of Agriculture 2007/2008, Small Holder Agriculture, Volume II: Crop Sector – National Report, Ministry of Agriculture, Food Security and Cooperatives; Ministry of Livestock Development and Fisheries; Ministry of Water and Irrigation; Ministry of Agriculture, Livestock and Environment, Zanzibar; Prime Minister’s Office; Regional Administration and Local Governments; Ministry of Industries, Trade and Marketing; The National Bureau of Statistics and the Office of the Chief Government Statistician, Zanzibar.

[cit0025] Westengen O.T. and Brysting A.K. (2014), “Crop adaptation to climate change in the semi-arid zone in Tanzania: the role of genetic resources and seed systems’, agriculture and”, Agriculture and Food Security, Vol. 3 No. 1, pp. 3-12.

